# Clinical significance of presystolic wave attenuation in patients with paroxysmal atrial fibrillation

**DOI:** 10.1007/s10840-026-02352-4

**Published:** 2026-05-14

**Authors:** Emrah Bayam, Macit Kalçık, Ersin Yıldırım, Burak Öztürkeri, Muzaffer Kahyaoğlu, Ayhan Küp, Baver Bozan, Mustafa Kara, Abdullah Sarıhan, Tolga Aksu, Ramazan Kargın

**Affiliations:** 1 Department of Cardiology, University of Medical Sciences, Kosuyolu High Specialty Training and Research Hospital, İstanbul, Turkey; 2 Department of Cardiology, Faculty of Medicine, Hitit University, Çorum, Turkey; 3 Department of Cardiology, Sancaktepe Prof. Dr. İlhan Varank Training and Research Hospital, İstanbul, Turkey; 4 Department of Cardiology, Ümraniye Training and Research Hospital, İstanbul, Turkey; 5 Department of Cardiology, Faculty of Medicine, İstinye University, İstanbul, Turkey

**Keywords:** Presystolic wave, Paroxysmal atrial fibrillation, Cerebrovascular events, Echocardiography, Atrial mechanical function

## Abstract

**Background:**

The presystolic wave (PSW), a late-diastolic Doppler flow signal in the left ventricular outflow tract, reflects atrial contraction and atrioventricular coupling. Although its presence is considered a physiological finding in healthy individuals, alterations in PSW characteristics may indicate impaired atrial or ventricular compliance. This study aimed to evaluate the relationship between PSW and cerebrovascular events (CVE) in patients with paroxysmal atrial fibrillation (PAF).

**Methods:**

In this retrospective study, 380 patients diagnosed with PAF by 24-hour Holter monitoring between January 2019 and January 2025 were analyzed. Transthoracic echocardiography was performed to assess PSW presence, left ventricular systolic and diastolic functions. Patients were divided according to PSW presence and history of CVE (stroke or transient ischemic attack). Clinical, laboratory, and echocardiographic variables were compared, and multivariate logistic regression was used to identify independent predictors of CVE.

**Results:**

PSW was identified in 284 (74.7%) patients. Those without PSW were older, had greater left atrial volume index (LAVI) and left ventricular mass index (LVMI), and more frequently had previous CVE (45.8% vs. 16.9%, *p* < 0.001). In multivariate analysis, high CHA₂DS₂-VASc score, increased LAVI, and absence of PSW were independent predictors of CVE. ROC analysis showed that a PSW peak velocity < 0.30 m/s was associated with the presence of CVE, with 81% sensitivity and 67% specificity (AUC = 0.783, *p* < 0.001).

**Conclusion:**

Absence or reduction of the PSW is independently associated with CVE in PAF patients. PSW assessment provides a simple, reproducible, and noninvasive measure of atrioventricular mechanical function that may be associated with increased thromboembolic vulnerability.

**Graphical Abstract:**

**Figure:**
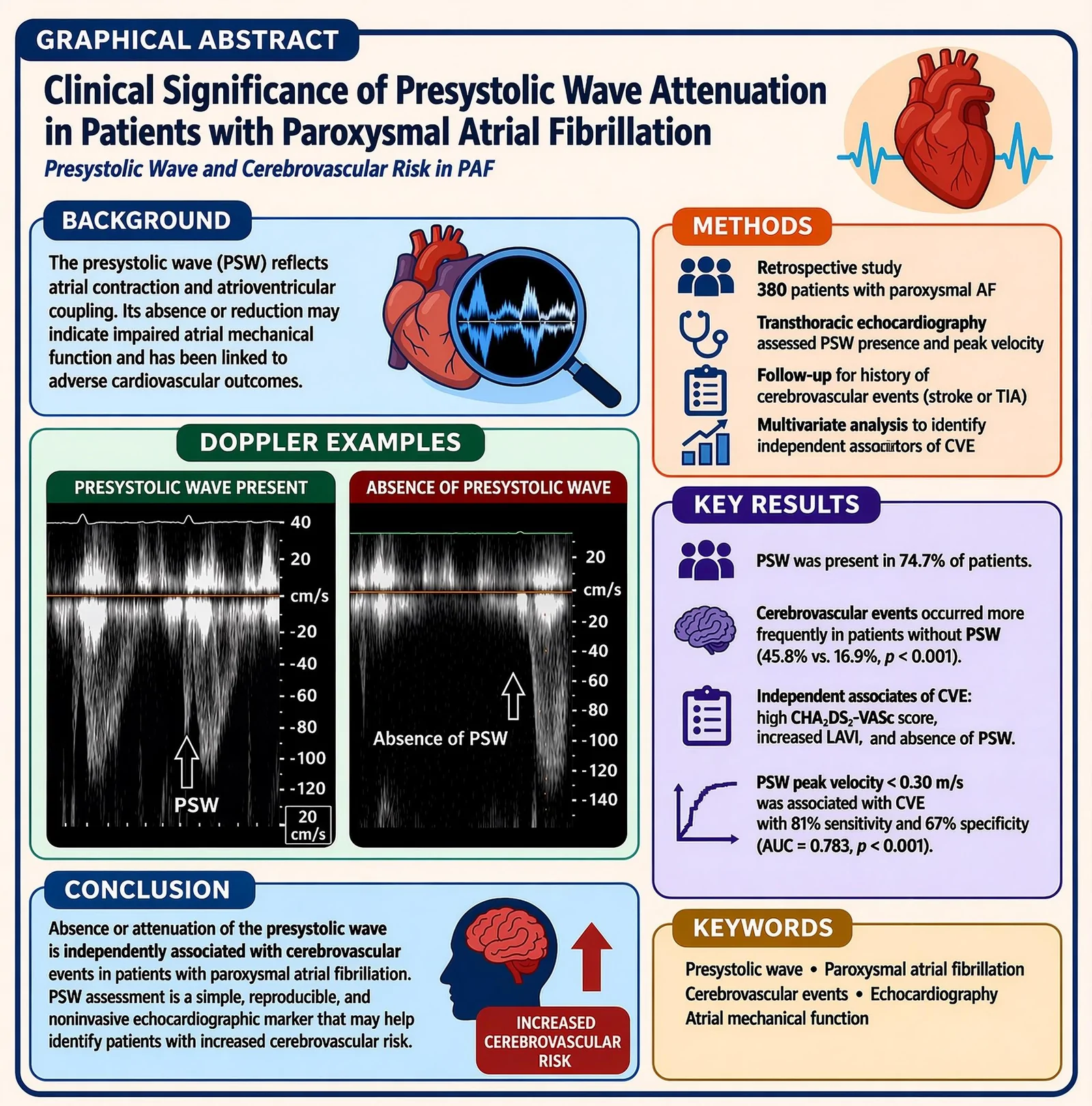
This study demonstrates that attenuation or absence of the presystolic wave (PSW) in the left ventricular outflow tract is associated with cerebrovascular events in patients with paroxysmal atrial fibrillation (PAF). PSW reduction reflects impaired atrioventricular coupling related to left ventricular hypertrophy, diastolic dysfunction, and reduced left atrial contractile function. Decreased PSW peak velocity and velocity–time integral (VTI) may indicate subclinical atrial mechanical dysfunction. PSW assessment by routine echocardiography may provide a simple and reproducible functional marker associated with cerebrovascular involvement in patients with PAF and may offer complementary information alongside conventional clinical risk assessment.

## Introduction

Atrial fibrillation (AF) is the most common sustained cardiac arrhythmia encountered in clinical practice and represents a major cause of morbidity and mortality due to its association with thromboembolic complications, particularly ischemic stroke [1]. Paroxysmal atrial fibrillation (PAF), characterized by self-terminating episodes of arrhythmia, shares a similar thromboembolic risk with persistent or permanent AF, despite its transient nature [2, 3]. The mechanisms underlying this risk extend beyond rhythm disturbance and involve structural, mechanical, and endothelial alterations within the atria and left ventricle [4]. Identification of echocardiographic markers that reflect atrial mechanical dysfunction has therefore become a major area of interest to improve risk stratification for cerebrovascular events (CVE) in patients with PAF.

The presystolic wave (PSW), a low-amplitude Doppler flow signal detected just before systolic ejection in the left ventricular outflow tract (LVOT), represents an atrial contraction–related component of left ventricular filling [5]. The PSW occurs during late diastole and is closely related to atrial mechanical function and left ventricular compliance [6]. Under normal physiological conditions, the presence of PSW indicates preserved atrioventricular coupling and effective atrial contribution to ventricular preload. However, PSW amplitude and velocity may diminish or disappear in conditions characterized by impaired atrial contractility, reduced left ventricular compliance, or advanced diastolic dysfunction [7, 8]. Consequently, PSW assessment has been proposed as a noninvasive surrogate of atrial–ventricular mechanical interaction.

Structural cardiac remodeling, including left ventricular hypertrophy (LVH), left atrial enlargement, and diastolic dysfunction, contributes to the deterioration of atrial mechanical performance and increases thromboembolic risk [9, 10]. As these abnormalities progress, PSW may attenuate or vanish, reflecting the loss of effective atrial contraction and late diastolic flow. Given that atrial mechanical failure is a critical determinant of stasis and thrombus formation, it is plausible that the absence or reduction of PSW velocity could be associated with CVE in PAF patients [11].

Based on this pathophysiological rationale, the present study aimed to investigate the relationship between the presence and characteristics of the PSW and the occurrence of CVE in patients with PAF. We hypothesized that PSW represents a marker of favorable atrioventricular mechanical function and that its absence or reduced velocity may be associated with impaired diastolic filling, atrial dysfunction, and a higher risk of stroke or transient ischemic attacks in PAF patients.

## Materials and methods

### Study design and population

This retrospective observational study included patients who underwent 24-hour rhythm Holter monitoring at the cardiology outpatient clinic between January 2019 and January 2025. Among these, individuals diagnosed with PAF were identified and enrolled. Patients with incomplete echocardiographic or clinical data were excluded. Additional exclusion criteria included moderate to severe native valvular stenosis or insufficiency, prosthetic heart valves, congestive heart failure, persistent or permanent atrial fibrillation, congenital heart disease, previous myocardial infarction or coronary artery bypass grafting, hypertrophic obstructive cardiomyopathy, infective endocarditis, metabolic or electrolyte disorders, and known significant coronary artery disease. Patients with non-critical coronary artery lesions documented by angiography or computed tomography were not excluded. Demographic and clinical characteristics, including comorbidities and medication history, were obtained retrospectively from hospital electronic medical records. In addition, a proportion of patients included in the study population were referred for cardiology evaluation following neurological consultation for suspected or confirmed cerebrovascular events, which contributed to the relatively higher prevalence of CVE observed in this cohort.

The presence of a previous CVE was defined as a documented history of ischemic stroke or transient ischemic attack confirmed by neurological evaluation and/or neuroimaging. Therefore, the study population partly represents a clinically enriched cohort referred for combined cardiology and neurology assessment rather than a purely screening-based PAF population. Patients receiving ongoing anticoagulant therapy before Holter evaluation were excluded to avoid confounding effects on thromboembolic risk assessment. The study protocol was approved by the institutional ethics committee, and all procedures conformed to the principles of the Declaration of Helsinki. Written informed consent was obtained from all participants prior to inclusion.

## Diagnosis of PAF

PAF was diagnosed based on 24-hour ambulatory rhythm Holter monitoring recordings. All Holter data were independently reviewed by two experienced cardiologists who were blinded to the echocardiographic findings. PAF was defined as episodes of AF lasting ≥ 30 s, characterized by absence of distinct P waves, irregular R-R intervals, and fibrillatory baseline activity, with spontaneous termination within 7 days. In cases of diagnostic disagreement, a consensus decision was reached through joint re-evaluation. Interobserver agreement for the detection of PAF episodes was high (κ > 0.85), indicating excellent reproducibility. Patients with only premature atrial contractions, or other sustained supraventricular arrhythmias were excluded. PAF was diagnosed during cardiology evaluation using 24-hour Holter monitoring, and therefore AF detection did not necessarily precede the cerebrovascular event in all patients.

## Laboratory analysis

Venous blood samples were obtained from all participants following an overnight fast on the morning of or within 24 h of Holter monitoring. Complete blood count (CBC) and biochemical analyses were performed using standard automated analyzers (Beckman Coulter LH-750 for hematologic parameters and Beckman Coulter L×20 for biochemical measurements). The following laboratory parameters were recorded for each patient: white blood cell (WBC) count, hemoglobin, platelet count, fasting glucose, blood urea nitrogen (BUN), creatinine, sodium, potassium, alanine aminotransferase (ALT), aspartate aminotransferase (AST), low-density lipoprotein (LDL), high-density lipoprotein (HDL), triglycerides, total cholesterol, and C-reactive protein (CRP). All tests were conducted in the hospital’s central biochemistry laboratory according to standardized protocols. Laboratory data were retrieved from electronic medical records and included the most recent measurements available prior to Holter monitoring.

## Echocardiographic Evaluation

Transthoracic echocardiographic examinations were performed for all study participants using a Vivid 5 system (GE Vingmed Ultrasound AS, Horten, Norway) equipped with a 2.5-MHz phased-array transducer. All measurements were obtained by two experienced cardiologists who were blinded to patients’ clinical and Holter findings. Standard B-mode, M-mode, and Doppler evaluations were conducted in accordance with current guidelines [12]. In patients with a history of CVE, transthoracic echocardiography was performed at least three months after the acute event during a clinically stable period.

Left ventricular ejection fraction (LVEF) was calculated using the biplane Simpson’s method. Left atrial volume was measured using the biplane area–length method from apical four- and two-chamber views at ventricular end-systole in accordance with current echocardiographic guidelines. The left atrial volume was then indexed to body surface area to obtain the left atrial volume index (LAVI). Right ventricular systolic function was assessed by tricuspid annular plane systolic excursion (TAPSE) on M-mode imaging. Left ventricular end-diastolic diameter (LVEDD) and end-systolic diameter (LVESD) were measured from the parasternal long-axis view using M-mode imaging at the level of the mitral valve leaflet tips. Interventricular septal thickness (IVST) and posterior wall thickness (PWT) were obtained at end-diastole. These measurements were used to calculate left ventricular mass (LVM) according to the Devereux formula, and the result was indexed to body surface area to determine the left ventricular mass index (LVMI). The presence of LVH was defined based on sex-specific LVMI thresholds (≥ 115 g/m² for men and ≥ 95 g/m² for women) [12].

Transmitral inflow velocities, including early (E) and late (A) diastolic peak velocities were measured by pulsed-wave Doppler in the apical four-chamber view. The E/A ratio was calculated to assess left ventricular diastolic filling patterns. Tissue Doppler imaging was used to obtain early diastolic (e’) and late diastolic (a’) mitral annular velocities, and the E/e’ ratio was calculated to estimate left ventricular filling pressures. Isovolumic relaxation time (IVRT) and deceleration time (DT) were also measured for diastolic assessment.

**Fig. 1:**
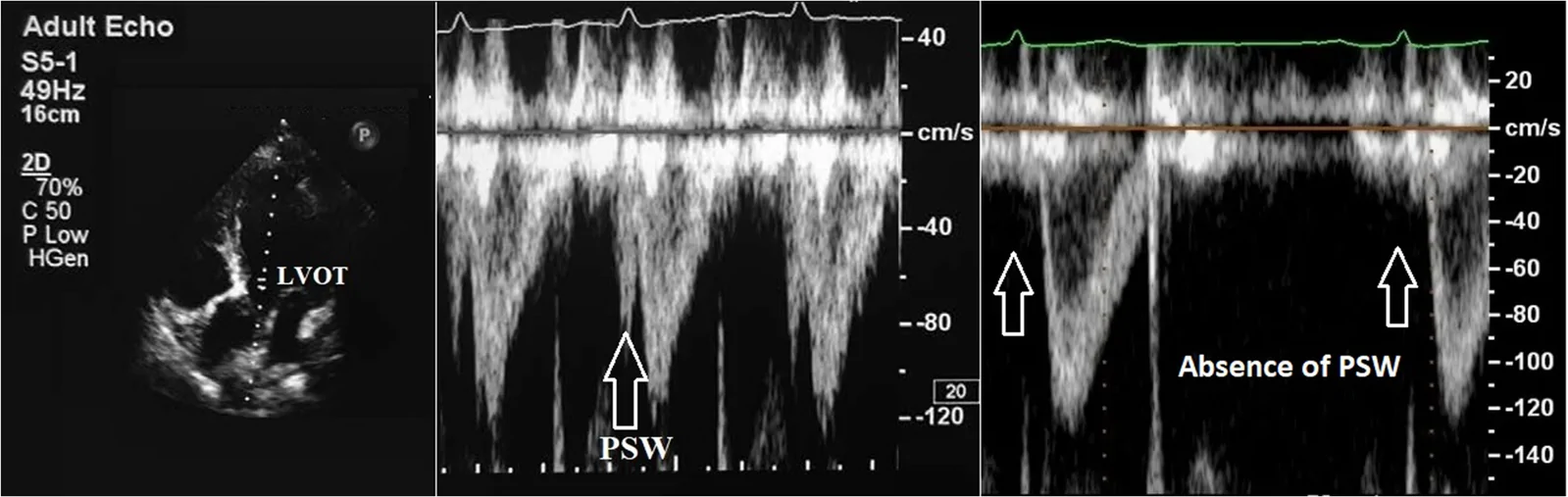
Representative pulsed-wave Doppler evaluation of the left ventricular outflow tract (LVOT) obtained from the apical five-chamber view. The left panel demonstrates sample volume positioning within the LVOT. The middle panel shows a typical Doppler tracing with a clearly visible presystolic wave (PSW) appearing as a low-amplitude late-diastolic flow signal immediately preceding systolic ejection (arrow). The right panel illustrates a tracing with absence of PSW despite adequate Doppler alignment, highlighted by arrows, indicating loss of late-diastolic atrial-related flow

Doppler evaluation of the LVOT was performed in the apical five-chamber view, positioning the pulsed Doppler sample volume just below the aortic valve on the ventricular side. The peak LVOT flow velocity and the velocity-time integral (VTI) of systolic ejection were recorded. Particular attention was paid to the presence of a PSW, defined as a low-amplitude flow signal occurring immediately before the onset of systolic LVOT flow and synchronous with late diastolic atrial contraction (the A-wave). When a measurable PSW signal was observed, both PSW peak velocity (m/s) and PSW VTI (cm) were quantified (Fig. 1). All Doppler measurements were averaged over three consecutive cardiac cycles at end-expiration during sinus rhythm. All Doppler gain, sweep speed, and filter settings were optimized according to standard echocardiographic protocols to ensure consistent PSW visualization. Particular care was taken to ensure optimal Doppler alignment with LVOT flow, and PSW measurements were performed only during sinus rhythm to minimize rhythm-related variability. The echocardiographer performing these measurements was blinded to all clinical and Holter data, ensuring unbiased evaluation.

PSW presence and peak velocity measurements were independently evaluated by two experienced echocardiographers who were blinded to clinical data. To assess intra-observer variability, measurements were performed by the same observer in a subset of randomly selected patients at different time points. Inter-observer agreement for PSW detection was assessed using Cohen’s kappa coefficient, and reproducibility of PSW peak velocity measurements was evaluated using intraclass correlation coefficients (ICC). Intra- and inter-observer agreement for PSW detection was excellent (κ > 0.85), and ICC values for PSW peak velocity measurements demonstrated high reproducibility (ICC > 0.90).

The CHA₂DS₂-VASc score was calculated for each patient based on age, sex, hypertension, diabetes mellitus, history of cerebrovascular events, and presence of vascular disease. This score was included in the multivariate logistic regression model to adjust for established clinical thromboembolic risk factors and to evaluate the incremental predictive value of PSW parameters beyond conventional risk stratification.

### Statistical analysis

All statistical analyses were performed using IBM SPSS Statistics for Windows, version 19.0 (IBM Corp., Armonk, NY, USA). Continuous variables were expressed as mean ± standard deviation (SD) or median (interquartile range) according to data distribution, while categorical variables were presented as numbers and percentages. The Kolmogorov–Smirnov test was used to evaluate normality. For group comparisons, the Student’s t-test was applied to normally distributed continuous variables and the Mann–Whitney U test to non-normally distributed ones. Chi-square or Fisher’s exact tests were used for categorical data analysis. Significant variables in univariate analyses were entered into a multivariate logistic regression model to identify independent predictors of CVE. In cases where variables were highly correlated with each other, only the one with the strongest clinical relevance or statistical association was included in the final model to avoid multicollinearity. Results were expressed as odds ratios (OR) with 95% confidence intervals (CI). The model’s calibration was assessed using the Hosmer–Lemeshow test, and overall model fit was expressed by Nagelkerke’s R². Receiver operating characteristic (ROC) analysis was performed to assess the predictive performance of LAVI and PSW peak velocity for CVE. The area under the curve (AUC), optimal cutoff values, and corresponding sensitivity and specificity were calculated using the Youden index. A two-tailed *p* value < 0.05 was considered statistically significant.

## Results

A total of 380 patients who were diagnosed with PAF on 24-hour rhythm Holter monitoring were included in the final analysis. Among the entire study population, a presystolic wave (PSW) could be clearly identified in 284 patients (74.7%), while 96 patients (25.3%) demonstrated an absence of PSW on Doppler echocardiography. Patients were stratified into two main groups according to the presence or absence of PSW.

The mean age was significantly higher in patients without PSW compared to those with PSW (59.7 ± 9.8 vs. 57.1 ± 11.8 years, *p* = 0.037). The distribution of sex, hypertension, diabetes mellitus, and dyslipidemia did not differ between groups (*p* > 0.05). However, smoking was more frequent in patients without PSW (38.5% vs. 23.9%, *p* = 0.006). The prevalence of previous CVE was also markedly higher in the PSW(–) group (45.8% vs. 16.9%, *p* < 0.001). The CHA₂DS₂-VASc score was comparable between patients with and without PSW, indicating similar baseline clinical thromboembolic risk profiles between the groups. Laboratory findings, including CBC parameters, glucose, lipid profile, liver enzymes, renal function, and C-reactive protein, were comparable between groups (*p* > 0.05). No significant difference was found in inflammatory or metabolic parameters (Table 1).

**Table 1:** The comparison of demographic, laboratory and echocardiographic parameters between paroxysmal AF patients with and without presystolic wave (PSW)

Variables	All Patients (*n* = 380)	PSW (+) (*n* = 284)	PSW (-) (*n* = 96)	*p* value
Demographic Parameters				
Age (years)	57.8 ± 11.4	57.1 ± 11.8	59.7 ± 9.8	**0.037**
Gender, male, n (%)	224 (58.9)	172 (60.6)	52 (54.2)	0.271
Hypertension, n (%)	168 (44.2)	128 (45.1)	40 (41.7)	0.562
Diabetes Mellitus, n (%)	60 (15.8)	48 (16.9)	12 (12.5)	0.307
Dyslipidemia, n (%)	62 (16.3)	44 (15.5)	18 (18.8)	0.455
Smoking status, n (%)	105 (27.6)	68 (23.9)	37 (38.5)	**0.006**
Non-obstructive CAD, n(%)	42 (11.1)	29 (10.2)	13 (13.5)	0.368
BMI, (kg/m^2^)	27.3 ± 3.4	27.2 ± 3.6	27.4 ± 2.5	0.829
Previous CVE, n (%)	92 (24.2)	48 (16.9)	44 (45.8)	**< 0.001**
CHA₂DS₂-VASc Score	4 (2–6)	4 (2–6)	3 (2–5)	0.346
Laboratory Parameters				
WBC (×10³/µL)	8.67 ± 2.6	8.6 ± 2.8	8.8 ± 2.3	0.717
Hemoglobin (g/dL)	13.1 ± 1.8	13.1 ± 1.8	13.2 ± 2.1	0.922
Platelet (×10³/µL)	247.7 ± 75.6	249.1 ± 80.8	243.6 ± 58.1	0.542
Glucose (mg/dL)	124.9 ± 55.7	129.1 ± 57.6	112.9 ± 49.7	0.199
BUN (mg/dL)	18.5 ± 7.1	18.7 ± 7.3	17.8 ± 6.1	0.293
Creatinine (mg/dL)	0.88 (0.77–1.1)	0.90 (0.79–1.2)	0.81 (0.75–1.1)	0.587
Sodium (mmol/L)	137.8 ± 4.2	138.1 ± 4.1	137.5 ± 3.9	0.318
Potassium (mmol/L)	4.39 ± 0.36	4.38 ± 0.36	4.41 ± 0.38	0.638
ALT (U/L)	20.6 ± 8.7	21.1 ± 8.9	20.4 ± 8.1	0.251
AST (U/L)	21.5 ± 9.3	21.8 ± 10.2	20.4 ± 7.1	0.244
Low-density lipoprotein (mg/dL)	106.2 ± 38.5	105.1 ± 37.5	108.3 ± 41.5	0.619
High-density lipoprotein (mg/dL)	38.7 ± 12.6	38.9 ± 13.4	38.2 ± 10.1	0.655
Triglyceride (mg/dL)	146.8 ± 80.7	144.4 ± 79.9	153.7 ± 82.8	0.375
Total cholesterol (mg/dL)	173.6 ± 42.8	171.5 ± 42.5	179.4 ± 43.6	0.167
CRP (mg/L)	5.9 (1.7–12)	4.7 (1.3–11.3)	7.8 (3.1–12.1)	0.102
Echocardiographic Parameters				
LVEF, (%)	64 ± 3.5	64.1 ± 3.3	63.5 ± 4.2	0.197
LVEDD, (mm)	45.3 ± 4.7	45.2 ± 4.9	45.7 ± 3.9	0.375
LVESD, (mm)	28.6 ± 3.3	28.5 ± 3.5	29.1 ± 2.8	0.118
IVST, (mm)	9.2 ± 0.9	9.1 ± 1.1	9.3 ± 0.9	0.138
PWT, (mm)	9.4 ± 1	9.3 ± 1.1	9.5 ± 0.9	0.144
LVM, (g)	146.5 ± 39.1	144.5 ± 38.7	152.6 ± 39.5	0.084
LVMI, (g/m^2^)	77.9 ± 22.5	76.4 ± 21.7	82.4 ± 24.3	**0.025**
LVH, n(%)	68 (17.9)	40 (14.1)	28 (29.2)	**0.001**
LAVI, (mL/m²)	27.9 ± 3.8	27.6 ± 3.6	28.9 ± 4.2	**0.012**
TAPSE, (cm)	2.13 ± 0.35	2.16 ± 0.36	2.05 ± 0.31	**0.004**
E, (m/s)	0.84 ± 0.21	0.86 ± 0.19	0.79 ± 0.21	**0.007**
A, (m/s)	0.82 ± 0.24	0.83 ± 0.25	0.76 ± 0.21	**0.015**
E/A ratio	1.14 ± 0.45	1.13 ± 0.45	1.15 ± 0.46	0.741
e’, (cm/s)	11.4 ± 3.6	11.6 ± 3.6	10.9 ± 3.6	0.083
a’, (cm/s)	10.7 ± 3.1	10.9 ± 3.2	10.3 ± 2.8	0.087
e’/a’ ratio	1.19 ± 0.58	1.19 ± 0.59	1.17 ± 0.55	0.735
E/e’ ratio	9.5 ± 1.7	8.6 ± 1.2	12.1 ± 2.3	**0.015**
DT, (ms)	167.1 ± 45.6	170.2 ± 46.7	157.6 ± 40.9	**0.019**
IVRT, (ms)	114.8 ± 15.7	113.5 ± 14.7	118.7 ± 17.7	**0.005**
PSW Peak Velocity, (m/s)	0.45 ± 0.19	0.45 ± 0.19	-	-
PSW VTI, (cm)	4.8 ± 2.2	4.8 ± 2.2	-	-
LVOT Peak Velocity, (m/s)	1.24 ± 0.29	1.24 ± 0.33	1.25 ± 0.19	0.885
LVOT VTI, (cm)	23.6 ± 4.87	23.1 ± 4.8	24.7 ± 4.4	0.142

*AF* Atrial fibrillation, *ALT* Alanine aminotransferase, *AST* Aspartate aminotransferase, *BMI* Body mass index, *BUN* Blood urea nitrogen, *CAD* Coronary artery disease, *CRP* C-reactive protein, *CVE* Cerebrovascular event, *DT* Deceleration time, *E* Early diastolic transmitral flow velocity, *A* Late diastolic transmitral flow velocity, *e*′, Early diastolic mitral annular velocity, *a*′, Late diastolic mitral annular velocity, *IVRT* Isovolumic relaxation time, *IVST* Interventricular septal thickness, *LVEF* Left ventricular ejection fraction, *LAVI* Left atrial volüme index, *LVH* Left ventricle hypertrophy, *LVEDD* Left ventricular end-diastolic diameter, *LVESD* Left ventricular end-systolic diameter, *LVOT* Left ventricular outflow tract, *PWT* Posterior wall thickness, *PSW* Presystolic wave, *TAPSE* Tricuspid annular plane systolic excursion, *VTI* Velocity time integral, *WBC* White blood cell count

**Fig. 2:**
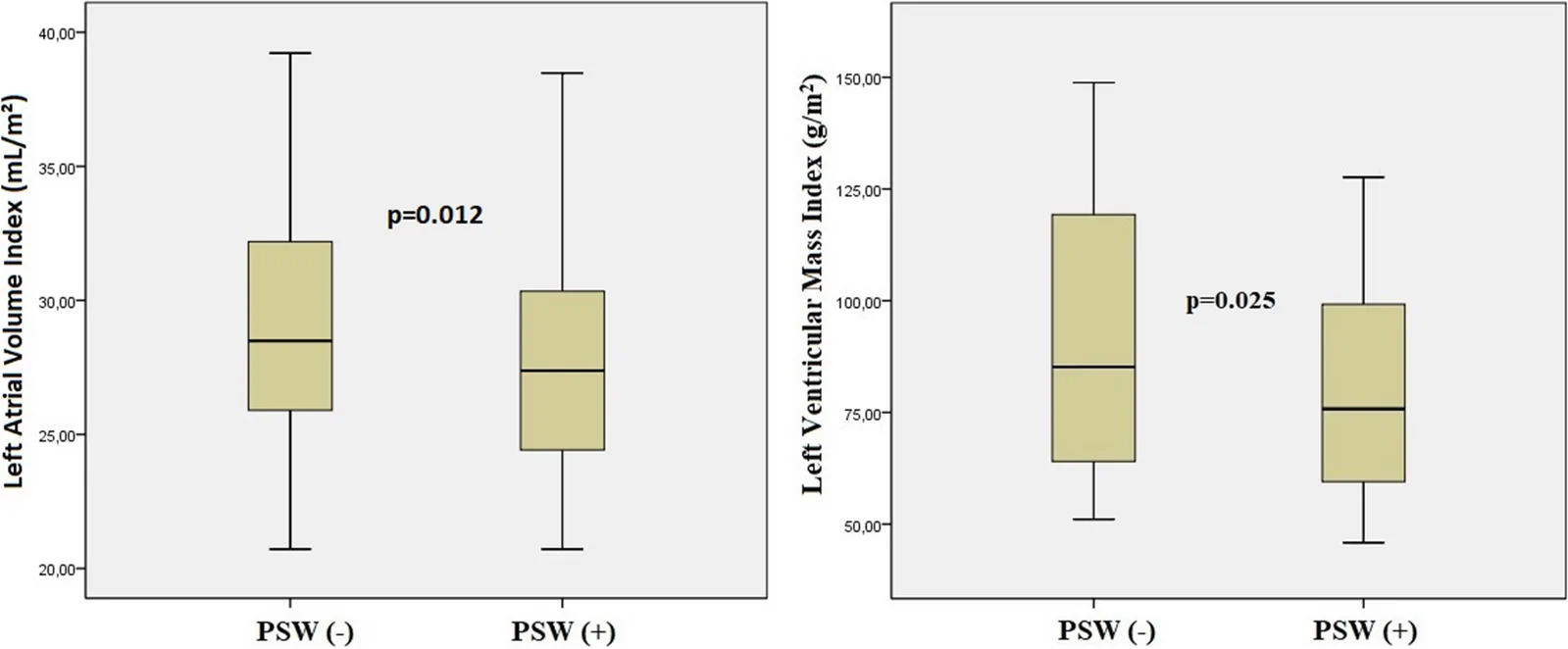
Comparison of structural echocardiographic parameters between patients with and without presystolic wave (PSW). Both left atrial volume index (LAVI) and left ventricular mass index (LVMI) were significantly higher in patients without PSW, indicating more advanced structural remodeling

Left ventricular systolic function, as assessed by left ventricular ejection fraction (LVEF), did not differ significantly between groups (64.1 ± 3.3% vs. 63.5 ± 4.2%, *p* = 0.197). Patients without PSW had a higher left ventricular mass index (LVMI) (82.4 ± 24.3 vs. 76.4 ± 21.7 g/m², *p* = 0.025) and a greater prevalence of left ventricular hypertrophy (LVH) (29.2% vs. 14.1%, *p* = 0.001). Similarly, LAVI was higher in the PSW(–) group (28.9 ± 4.2 vs. 27.6 ± 3.6 mL/m², *p* = 0.012) (Fig. 2). Patients with PSW demonstrated better right ventricular systolic function, reflected by higher TAPSE (2.16 ± 0.36 vs. 2.05 ± 0.31 cm, *p* = 0.004). In diastolic evaluation, early mitral inflow velocity (E) and late velocity (A) were both higher in the PSW(+) group (*p* = 0.007 and *p* = 0.015, respectively). The E/e′ ratio was significantly elevated in the PSW(–) group (12.1 ± 2.3 vs. 8.6 ± 1.2, *p* = 0.015), indicating higher left ventricular filling pressures. Furthermore, DT and IVRT were prolonged in patients without PSW (*p* = 0.019 and *p* = 0.005, respectively), suggesting impaired relaxation pattern (Table 1).

Patients were divided into two groups according to the presence or absence of a history of CVE. Among 380 patients with PAF, 92 patients (24.2%) had a documented history of stroke or transient ischemic attack (CVE group), while 288 patients (75.8%) had no prior cerebrovascular event (non-CVE group). Patients in the CVE(+) group were significantly older than those without CVE (64.6 ± 9.2 vs. 55.5 ± 11.1 years, *p* < 0.001). The prevalence of hypertension was markedly higher in patients with CVE (87% vs. 45.8%, *p* < 0.001), while the rates of diabetes mellitus, dyslipidemia, and smoking were comparable between groups (*p* > 0.05). The body mass index (BMI) was also higher in the CVE(+) group (28.8 ± 2.7 vs. 26.8 ± 3.4 kg/m², *p* < 0.001), indicating an association between increased adiposity and cerebrovascular risk. Patients with CVE had significantly higher CHA₂DS₂-VASc scores compared with those without CVE, reflecting a greater burden of established clinical thromboembolic risk factors. Laboratory findings, including glucose, renal function, and lipid profile, did not differ significantly between the groups (*p* > 0.05). Echocardiographic assessment revealed marked structural and functional differences. The LAVI was significantly greater in patients with CVE (29.8 ± 4.1 vs. 27.4 ± 3.5 mL/m², *p* < 0.001), suggesting more advanced atrial remodeling (Fig. 2). Similarly, LVMI and the prevalence of LVH were higher among those with CVE (85.8 ± 16.4 g/m² vs. 75.4 ± 21.3 g/m², *p* = 0.035; and 26.1% vs. 15.3%, *p* = 0.019, respectively). Both IVST and PWT were also significantly increased in the CVE(+) group indicating greater concentric remodeling of the left ventricle. Of particular importance, the presence of PSW was significantly less frequent among patients with CVE (52.2% vs. 81.9%, *p* < 0.001). Moreover, PSW peak velocity and PSW VTI were both substantially lower in the CVE(+) group (0.31 ± 0.13 vs. 0.48 ± 0.19 m/s, *p* < 0.001; and 4.27 ± 1.66 vs. 4.96 ± 2.29 cm, *p* = 0.017, respectively) (Table 2; Fig. 2).

**Table 2:** The comparison of demographic, laboratory and echocardiographic parameters between paroxysmal AF patients with and without cerebrovascular events (CVE)

Variables	CVE (+) (*n* = 92)	CVE (-) (*n* = 288)	*p* value
Demographic Parameters			
Age (years)	64.6 ± 9.2	55.5 ± 11.1	**< 0.001**
Gender, male, n (%)	48 (52.2)	176 (61.1)	0.129
Hypertension, n (%)	80 (87)	132 (45.8)	**< 0.001**
Diabetes Mellitus, n (%)	12 (13)	48 (16.7)	0.407
Dyslipidemia, n (%)	13 (14.1)	49 (17)	0.515
Smoking status, n (%)	21 (22.8)	84 (29.2)	0.236
Non-obstructive CAD, n(%)	13 (14.1)	29 (10.1)	0.279
BMI, (kg/m^2^)	28.8 ± 2.7	26.8 ± 3.4	**< 0.001**
CHA₂DS₂-VASc Score	5 (3–7)	3 (2–5)	**< 0.001**
Laboratory Parameters			
WBC (×10³/µL)	9.3 ± 3.1	8.5 ± 2.5	0.107
Hemoglobin (g/dL)	13.2 ± 1.9	13.1 ± 1.8	0.643
Platelet (×10³/µL)	248.7 ± 71.3	247.4 ± 77.1	0.879
Glucose (mg/dL)	125.6 ± 43.2	124.7 ± 33.4	0.946
BUN (mg/dL)	18.3 ± 7.4	18.6 ± 6.9	0.678
Creatinine (mg/dL)	0.86 (0.78–1.1)	0.9 (0.77–1.1)	0.451
Sodium (mmol/L)	137.8 ± 2.4	136.3 ± 2.1	0.748
Potassium (mmol/L)	4.39 ± 0.37	4.35 ± 0.36	0.729
ALT (U/L)	20.4 ± 11.6	20.8 ± 12	0.785
AST (U/L)	21.4 ± 10.8	21.5 ± 9.8	0.939
Low-density lipoprotein (mg/dL)	105.4 ± 43.5	106.5 ± 36.9	0.852
High-density lipoprotein (mg/dL)	39.1 ± 13.1	38.4 ± 12.5	0.676
Triglyceride (mg/dL)	154.8 ± 76.6	144.2 ± 64.9	0.329
Total cholesterol (mg/dL)	170.8 ± 47.4	174.5 ± 41.3	0.530
CRP (mg/L)	5.1 (1.9–12)	6.8 (1.5–12)	0.842
Echocardiographic Parameters			
LVEF, (%)	63.7 ± 4.3	64.1 ± 3.3	0.407
LVEDD, (mm)	44.5 ± 3.8	45.6 ± 4.9	**0.023**
LVESD, (mm)	27.9 ± 2.8	28.8 ± 3.4	**0.012**
IVST, (mm)	9.3 ± 1	8.9 ± 0.9	**0.001**
PWT, (mm)	9.5 ± 1	9.1 ± 0.9	**< 0.001**
LVM, (g)	153.1 ± 40.3	143.5 ± 34.4	0.095
LVMI, (g/m^2^)	85.8 ± 16.4	75.4 ± 21.3	**0.035**
LVH, n(%)	24 (26.1)	44 (15.3)	**0.019**
LAVI, (mL/m²)	29.8 ± 4.1	27.4 ± 3.5	**< 0.001**
TAPSE, (cm)	2.11 ± 0.31	2.15 ± 0.36	0.125
E, (m/s)	0.87 ± 0.23	0.84 ± 0.19	0.274
A, (m/s)	0.86 ± 0.24	0.82 ± 0.24	0.135
E/A ratio	1.11 ± 0.43	1.15 ± 0.46	0.361
e’, (cm/s)	11.7 ± 4.1	11.4 ± 3.5	0.498
a’, (cm/s)	11.3 ± 2.6	10.8 ± 3.2	0.123
e’/a’ ratio	1.11 ± 0.51	1.22 ± 0.6	0.081
E/e’ ratio	12.4 ± 5.6	8.6 ± 4.4	**0.009**
DT, (ms)	162.5 ± 36.1	168.5 ± 48.3	0.208
IVRT, (ms)	114.7 ± 19.1	113.8 ± 14.4	0.936
Presence of PSW, n(%)	48(52.2)	236 (81.9)	**< 0.001**
PSW Peak Velocity, (m/s)	0.31 ± 0.13	0.48 ± 0.19	**< 0.001**
PSW VTI, (cm)	4.27 ± 1.66	4.96 ± 2.29	**0.017**
LVOT Peak Velocity, (m/s)	1.22 ± 0.21	1.25 ± 0.82	0.691
LVOT VTI, (cm)	24.5 ± 4.3	23.7 ± 5.1	0.141

*AF* Atrial fibrillation, *ALT* Alanine aminotransferase, *AST* Aspartate aminotransferase, *BMI* Body mass index, *BUN* Blood urea nitrogen, *CAD* Coronary artery disease, *CRP* C-reactive protein, *CVE* Cerebrovascular event, *DT* Deceleration time, *E* Early diastolic transmitral flow velocity, *A* Late diastolic transmitral flow velocity, *e*′: Early diastolic mitral annular velocity, *a*′ Late diastolic mitral annular velocity, IVRT Isovolumic relaxation time, *IVST* Interventricular septal thickness, *LVEF* Left ventricular ejection fraction, *LAVI* Left atrial volüme index, *LVH* Left ventricle hypertrophy, *LVEDD* Left ventricular end-diastolic diameter, *LVESD* Left ventricular end-systolic diameter, *LVOT* Left ventricular outflow tract, *PWT* Posterior wall thickness, *PSW* Presystolic wave, *TAPSE* Tricuspid annular plane systolic excursion, *VTI* Velocity time integral, *WBC* White blood cell count

Variables found to be significant in univariate analysis were entered into a multivariate logistic regression model to identify independent predictors of CVE in patients with PAF. After adjustment for CHA₂DS₂-VASc score, structural remodeling parameters, diastolic burden, and smoking status; the CHA₂DS₂-VASc score (OR: 1.422; 95% CI: 1.180–1.715; *p* < 0.001), LAVI (OR: 1.095; 95% CI: 1.024–1.278; *p* = 0.011), and absence of PSW (OR: 1.881; 95% CI: 1.220–2.961; *p* = 0.004) remained independently associated with CVE (Table 3). In contrast, LVH, E/e′ ratio, and smoking status were not independently predictive after multivariable adjustment. These findings suggest that PSW attenuation may provide incremental information beyond established clinical risk stratification and echocardiographic markers of structural remodeling in patients with PAF, supporting the potential role of PSW as a novel marker of atrial mechanical dysfunction and thromboembolic vulnerability.

**Table 3:** The results for multivariate logistic regression analyses of univariate associates of cerebrovascular events in patients with paroxysmal atrial fibrillation

Parameters	Multivariate Analysis		
	OR	95% CI	*p* value
CHA₂DS₂-VASc score	1.422	1.180–1.715	**< 0.001**
Left Atrial Volume Index	1.095	1.024–1.278	**0.011**
Left Ventricle Hypertrophy	1.312	0.865–2.012	0.214
E/e′ ratio	1.156	0.964–1.328	0.116
Smoking	1.285	0.884–1.865	0.176
Absence of PSW	1.881	1.220–2.961	**0.004**

*CI *Confidence Interval, *OR* Odds Ratio, *PSW* Presystolic Wave

**Fig. 3:**
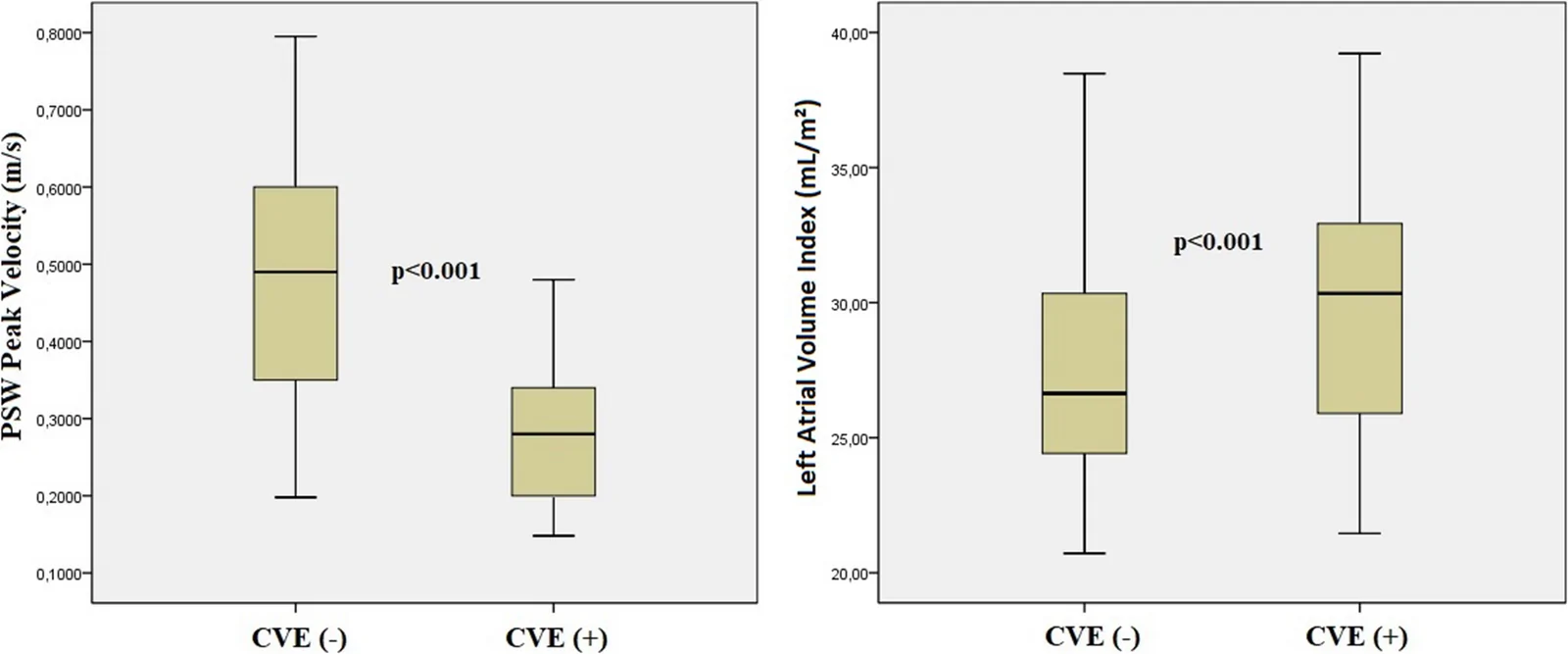
Comparison of presystolic wave (PSW) peak velocity and left atrial volume index (LAVI) according to cerebrovascular event (CVE) history. Patients with CVE showed markedly lower PSW peak velocity and higher LAVI, suggesting impaired atrioventricular mechanics and atrial enlargement as contributors to thromboembolic risk

**Fig. 4:**
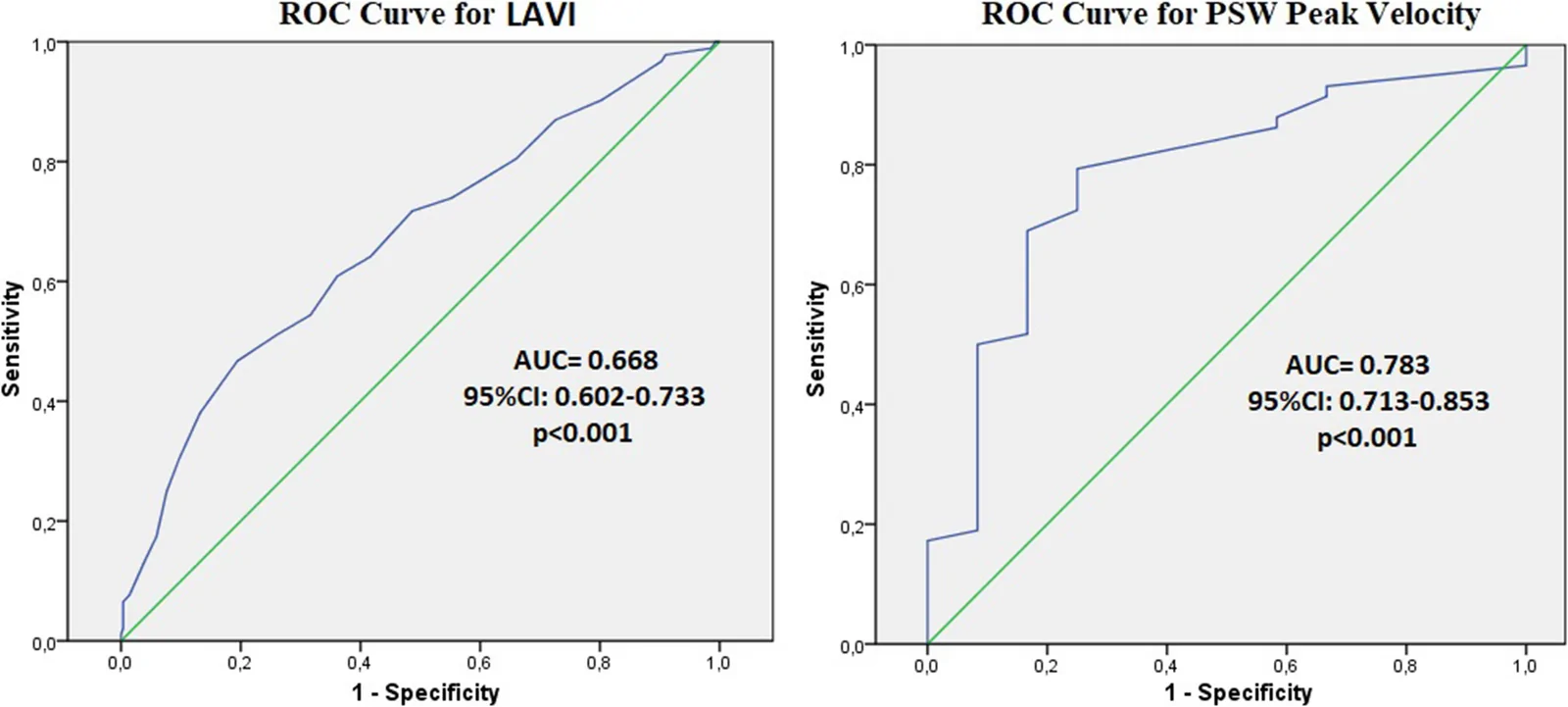
Receiver operating characteristic (ROC) analysis for prediction of cerebrovascular events. Both left atrial volume index (LAVI) and PSW peak velocity were significant predictors, with PSW velocity showing superior discriminative power (AUC = 0.783 vs. 0.688)

Receiver operating characteristic (ROC) curve analysis was performed to evaluate the discriminative ability of LAVI and PSW peak velocity for identifying patients with CVE. A LAVI above 28 mL/m² yielded a sensitivity of 64% and specificity of 59% (AUC = 0.668; 95% CI: 0.602–0.733; *p* < 0.001). Furthermore, a PSW peak velocity below 0.30 m/s demonstrated the highest discriminative ability for identifying patients with prior CVE, with a sensitivity of 81% and specificity of 67% (AUC = 0.783; 95% CI: 0.713–0.853; *p* < 0.001) (Fig. 3 and Fig. 4).

## Discussion

In this study, we investigated the relationship between the PSW detected in the LVOT and CVE in patients with PAF. Our main finding is that the absence or attenuation of PSW was independently associated with a higher prevalence of CVE, even after adjustment for the CHA₂DS₂-VASc score and echocardiographic parameters such as LAVI and LVH. In addition, reduced PSW peak velocity demonstrated the highest diagnostic accuracy among all echocardiographic parameters for identifying patients with prior CVE. These findings suggest that PSW may serve as a novel and easily obtainable echocardiographic indicator associated with atrial mechanical dysfunction and history of cerebrovascular events in PAF.

The PSW represents late diastolic flow generated by active atrial contraction into the left ventricular outflow tract and is commonly observed in healthy individuals. In normal populations, the PSW can be detected in approximately 70–90% of subjects with normal diastolic function and preserved atrioventricular coupling [13, 14]. Its presence reflects intact atrial contractility, compliant left ventricle, and efficient atrioventricular mechanical interaction. Conversely, previous studies have shown that the PSW becomes attenuated or disappears in conditions associated with hypertensive heart disease, diastolic dysfunction, or impaired atrial contractile reserve [5].

A major novel aspect of our study is the demonstration that the absence of PSW or reduction in its velocity is associated with CVE in PAF patients. The pathophysiological explanation for this finding likely involves atrial mechanical dysfunction. As atrial compliance and contractile function deteriorate due to hypertrophy, fibrosis, or diastolic dysfunction, effective atrial ejection during late diastole declines [15]. This mechanical impairment may lead to increased stasis within the left atrium and left atrial appendage, thereby predisposing to thrombus formation and embolic stroke [16]. Unlike conventional markers such as LAVI or LVMI, which reflect structural remodeling, PSW reflects dynamic mechanical coupling and may therefore provide additional insight into the functional component of atrial failure [5]. The independent association of PSW absence with CVE in our multivariate analysis supports this hypothesis. Although PSW attenuation is related to structural remodeling and diastolic dysfunction, its independent association with CVE after adjustment for these parameters suggests that it should not be interpreted solely as a surrogate of chamber enlargement or diastolic impairment.

While PSW is physiologically linked to atrial contraction and ventricular compliance, it should not be viewed solely as a surrogate of structural remodeling. In the present study, PSW attenuation remained independently associated with CVE even after adjustment for left atrial volume index and left ventricular hypertrophy. This suggests that PSW reflects functional atrioventricular mechanical impairment beyond conventional structural and diastolic markers that are already known to influence stroke risk. Importantly, in our multivariable model including established markers of structural remodeling (LAVI and LVH) and diastolic burden (E/e′ ratio), the absence of PSW remained independently associated with cerebrovascular events. This finding suggests that PSW reflects functional atrioventricular mechanical impairment beyond conventional structural and diastolic echocardiographic parameters.

The predictive value of PSW peak velocity for CVE in our ROC analysis was notable. A cutoff of 0.30 m/s identified patients with prior CVE with good sensitivity (81%) and specificity (67%), surpassing both LAVI and age. These results emphasize that reduced PSW velocity may signal early impairment of atrial-ventricular mechanics, even before marked chamber dilation or overt diastolic dysfunction becomes apparent. From a clinical standpoint, PSW assessment is noninvasive, reproducible, and easily obtainable from a standard apical five-chamber view, making it a potentially useful complementary parameter during echocardiographic evaluation of patients with PAF.

In the multivariate model incorporating established clinical thromboembolic risk through the CHA₂DS₂-VASc score together with structural and functional echocardiographic parameters, PSW attenuation remained independently associated with CVE. While higher CHA₂DS₂-VASc score and increased LAVI emerged as expected strong predictors of CVE, the persistent significance of PSW absence suggests that this Doppler parameter captures functional atrioventricular mechanical impairment beyond conventional clinical risk stratification and atrial remodeling. In contrast, LVH, diastolic burden assessed by E/e′ ratio, and smoking status did not retain independent associations after comprehensive adjustment, indicating that their effects may be mediated through global clinical risk and atrial structural changes. These findings support the incremental prognostic value of PSW assessment in patients with PAF. Therefore, PSW assessment may provide complementary functional information in addition to conventional structural echocardiographic markers when evaluating cerebrovascular involvement in patients with paroxysmal atrial fibrillation.

Despite similar CHA₂DS₂-VASc scores between patients with and without PSW, the prevalence of CVE differed markedly between these groups. This discrepancy may reflect the fact that the CHA₂DS₂-VASc score is based primarily on clinical risk factors and does not include functional markers of atrioventricular mechanical coupling. In contrast, PSW represents a Doppler-derived parameter reflecting atrial mechanical function and late diastolic ventricular filling. In addition, because PAF was diagnosed during cardiology evaluation based on 24-hour Holter monitoring, AF detection did not necessarily precede cerebrovascular events in all patients. Therefore, PSW attenuation may provide complementary functional information beyond conventional clinical risk stratification in patients with PAF.

Our findings align with previous evidence linking atrial mechanical dysfunction and thromboembolic risk in AF. Prior studies have demonstrated that decreased left atrial strain, reduced atrial appendage emptying velocity, and impaired diastolic indices are associated with increased stroke risk [17, 18]. PSW, by integrating atrial contraction and ventricular compliance, could represent a complementary parameter in this continuum. It may provide additional information beyond traditional structural and clinical predictors, particularly in patients without persistent AF or overt structural heart disease.

Importantly, PSW assessment should not be interpreted as an alternative to established clinical risk stratification tools such as the CHA₂DS₂-VASc score. Rather, PSW may provide complementary functional information reflecting atrioventricular mechanical coupling that is not captured by conventional clinical variables. In this context, PSW evaluation may be particularly relevant in patients with comparable clinical risk scores but differing degrees of atrial mechanical impairment. However, prospective studies are required to determine whether incorporation of PSW into routine echocardiographic assessment can meaningfully improve cerebrovascular risk stratification in patients with PAF.

Although the physiological basis of the PSW has been described in previous studies, its clinical implications in patients with PAF have not been clearly defined. To the best of our knowledge, this is the first study to specifically evaluate the association between PSW characteristics and CVE in patients with PAF. The present study extends existing knowledge by demonstrating an independent association between PSW attenuation and CVE in a PAF population. Unlike traditional structural markers such as left atrial enlargement or ventricular hypertrophy, PSW reflects dynamic atrioventricular mechanical coupling. By linking this functional Doppler parameter to thromboembolic outcomes, our findings suggest that PSW may represent a clinically relevant marker of atrial mechanical dysfunction beyond its previously recognized physiological significance.

It should be noted that the clinical interpretation of PSW is not uniform across all studies in the literature. While our findings suggest that the presence of PSW reflects preserved atrioventricular mechanical coupling, several studies have associated the detection of PSW with subclinical left ventricular dysfunction in conditions such as diabetes mellitus, hypothyroidism, and acute myocardial infarction [19–21]. This discrepancy likely reflects differences in study populations and methodological approaches, as PSW behavior may vary according to underlying diastolic properties and loading conditions.

Several limitations should be acknowledged. Because of the retrospective cross-sectional design of the present single-center study, the findings should be interpreted as demonstrating association rather than predictive capability regarding future CVEs. Although patients receiving anticoagulant therapy before Holter monitoring were excluded, silent embolic events or subclinical AF episodes may have influenced the results. The relatively high prevalence of CVE (24.2%) in our study group compared with general PAF populations may be explained by referral-related selection bias [22]. A proportion of patients were evaluated after neurological consultation for suspected or confirmed CVEs, resulting in a clinically enriched cohort with higher baseline cerebrovascular risk than a typical screening-based PAF population. Accordingly, the observed association between PSW attenuation and CVE should be interpreted within the context of this enriched-risk study population. The PSW peak velocity cut-off was derived from the same study cohort using ROC analysis and has not been validated in an independent population. Therefore, this threshold should be interpreted as exploratory and hypothesis-generating rather than definitive, and prospective studies with external validation cohorts are required before its routine clinical application can be considered. A further limitation of this study is the lack of left atrial and left ventricular strain analysis, which could not be performed because of the retrospective design and non-standardized image acquisition for speckle-tracking echocardiography. Future prospective studies incorporating strain imaging may help to better define the mechanistic relationship between PSW and atrial myopathy. Finally, prospective, longitudinal, and multicenter studies are warranted to determine whether PSW absence or reduced velocity predicts future CVE in patients with PAF.

In conclusion, the present study demonstrates that the absence or reduced velocity of the presystolic wave is independently associated with CVE in patients with PAF. The PSW appears to reflect preserved atrioventricular mechanical coupling and its loss may indicate advanced diastolic dysfunction, atrial mechanical impairment, and increased thromboembolic risk. Incorporating PSW evaluation into standard echocardiographic assessment may provide additional information regarding cerebrovascular risk profile in patients with PAF.

## Data Availability

Data of this manuscript can be inquired upon reasonable request to the corresponding author.

## References

[1] Van Gelder IC, Rienstra M, Bunting KV, et al. 2024 ESC Guidelines for the management of atrial fibrillation developed in collaboration with the European Association for Cardio-Thoracic Surgery (EACTS). Eur Heart J. 2024;45(36):3314–414. 10.1093/eurheartj/ehae176 39210723

[2] Ganesan AN, Shipp NJ, Brooks AG, et al. Long-term outcomes of catheter ablation of atrial fibrillation: a systematic review and meta-analysis. J Am Heart Assoc. 2013;2(2):e004549. 10.1161/JAHA.112.004549 PMC3647286 23537812

[3] Ding WY, Harrison S, Gupta D, Lip GYH, Lane DA. Stroke and Bleeding Risk Assessments in Patients With Atrial Fibrillation: Concepts and Controversies. Front Med (Lausanne). 2020;7:54. 10.3389/fmed.2020.00054 PMC7047213 32154260

[4] Goette A, Kalman JM, Aguinaga L, et al. EHRA/HRS/APHRS/SOLAECE expert consensus on atrial cardiomyopathies: definition, characterization, and clinical implication. Europace. 2016;18(10):1455–90. 10.1093/europace/euw161 PMC6392440 27402624

[5] Joshi KR, Kabirdas D, Romero-Corral A, Shah M, Figueredo VM, Pressman GS. Clinical significance of a presystolic wave on Doppler examination of the left ventricular outflow tract. Am J Cardiol. 2014;114(10):1599–602. 10.1016/j.amjcard.2014.08.048 25440501

[6] Akyüz AR, Turan T, Gürbak İ, Korkmaz L, Ağaç MT, Çelik Ş. The relationship between presystolic wave and subclinical left ventricular dysfunction in asymptomatic hypertensive patients. Blood Press Monit. 2016;21(5):277–81. 10.1097/MBP.0000000000000199 27223102

[7] Nagueh SF, Appleton CP, Gillebert TC, et al. Recommendations for the evaluation of left ventricular diastolic function by echocardiography. Eur J Echocardiogr. 2009;10(2):165–93. 10.1093/ejechocard/jep007 19270053

[8] Dokainish H. Left ventricular diastolic function and dysfunction: Central role of echocardiography. Glob Cardiol Sci Pract. 2015;2015:3. 10.5339/gcsp.2015.3 PMC4374097 25830147

[9] Tsang TS, Barnes ME, Bailey KR et al. Left atrial volume: important risk marker of incident atrial fibrillation in 1655 older men and women. Mayo Clin Proc. 2001;76(5):467–475. 10.4065/76.5.467 11357793

[10] Ganau A, Devereux RB, Roman MJ, et al. Patterns of left ventricular hypertrophy and geometric remodeling in essential hypertension. J Am Coll Cardiol. 1992;19(7):1550–8. 10.1016/0735-1097(92)90617-v 1534335

[11] Hays AG, Sacco RL, Rundek T, et al. Left ventricular systolic dysfunction and the risk of ischemic stroke in a multiethnic population. Stroke. 2006;37(7):1715–9. 10.1161/01.STR.0000227121.34717.40 PMC2677017 16741172

[12] Lang RM, Badano LP, Mor-Avi V, et al. Recommendations for cardiac chamber quantification by echocardiography in adults: an update from the American Society of Echocardiography and the European Association of Cardiovascular Imaging. Eur Heart J Cardiovasc Imaging. 2015;16(3):233–70. 10.1093/ehjci/jev014 25712077

[13] Mittal SR, Pancholi N. Left ventricular outflow tract presystolic flow velocity–another marker of left ventricular diastolic function. Int J Cardiovasc Imaging. 2002;18(4):249–56. 10.1023/a:1015504609150 12123317

[14] Cowie B, Costello B, Wright L, et al. Diastolic flow in the left ventricular outflow tract - A normal physiological and underappreciated echocardiographic finding. Australas J Ultrasound Med. 2022;25(3):137–41. 10.1002/ajum.12307 PMC9351428 35978728

[15] Kuppahally SS, Akoum N, Burgon NS, et al. Left atrial strain and strain rate in patients with paroxysmal and persistent atrial fibrillation: relationship to left atrial structural remodeling detected by delayed-enhancement MRI. Circ Cardiovasc Imaging. 2010;3(3):231–9. 10.1161/CIRCIMAGING.109.865683 20133512

[16] Burstein B, Nattel S. Atrial fibrosis: mechanisms and clinical relevance in atrial fibrillation. J Am Coll Cardiol. 2008;51(8):802–9. 10.1016/j.jacc.2007.09.064 18294563

[17] Leung DY, Boyd A, Ng AA, Chi C, Thomas L. Echocardiographic evaluation of left atrial size and function: current understanding, pathophysiologic correlates, and prognostic implications. Am Heart J. 2008;156(6):1056–64. 10.1016/j.ahj.2008.07.021 19032999

[18] Hoit BD. Left atrial size and function: role in prognosis. J Am Coll Cardiol. 2014;63(6):493–505. 10.1016/j.jacc.2013.10.055 24291276

[19] Saylik F, Akbulut T. The association of presystolic wave with subclinical left-ventricular dysfunction in patients with subclinical hypothyroidism. J Echocardiogr. 2022;20(2):97–105. 10.1007/s12574-021-00556-3. 34633627

[20] Dursun I, Kul S, Sahin S, Kalaycioglu E, Akyuz AR, Korkmaz L. Relation of presystolic wave on doppler examination to syntax score in patients with acute myocardial infarction. Int J Cardiovasc Imaging. 2018;34(4):569–76. 10.1007/s10554-017-1270-4 29124470

[21] Kul S, Dursun İ, Ayhan S, et al. Presystolic Wave is Associated with Subclinical Left Ventricular Dysfunction Assessed by Myocardial Performance Index in Type 2 Diabetes Mellitus. Arq Bras Cardiol. 2019;113(2):207–15. 10.5935/abc.20190134 PMC6777877 31365600

[22] Healey JS, Connolly SJ, Gold MR, et al. Subclinical atrial fibrillation and the risk of stroke. N Engl J Med. 2012;366(2):120–9. 10.1056/NEJMoa1105575 22236222

